# Myrmica elmesi (Hymenoptera, Formicidae) a new species from Himalaya

**DOI:** 10.3897/zookeys.124.1586

**Published:** 2011-08-18

**Authors:** Himender Bharti, Yash Paul Sharma

**Affiliations:** Department of Zoology, Punjabi University Patiala, India 147002

**Keywords:** Ants, taxonomy, *Myrmica elmesi*, new species, *pachei* group, ecology, Himalaya

## Abstract

*Myrmica elmesi* **sp. n.** is described from Himalaya. This species belongs to the *pachei* group of *Myrmica* speciesand is distinct from the species described in this group hitherto, which is represented by 14 species including three from Indian Himalaya. *Myrmica elmesi* is the fourth species of the diverse *pachei* group found in Himalaya; it was collected from the transitional zone and is described with notes on its ecology, this gains significance in the sense that ecology of most of the old world *Myrmica* is either unknown or poorly known.

## Introduction

Genus *Myrmica* in the old world is represented by 142 valid species. These species are well distributed in Palearctic zone and South-east Asian tropical and subtropical regions. The central Asian mountains which comprise Hindu Kush, Karakorum, South-western slope of Himalaya (Afghanistan, Pakistan, India, Nepal and Bhutan) comprise 33 species representing 7 species groups. 31 species (94%) are endemic to this region. The diversity of this region is quite interesting because the species of this region show a plesiomorphic state of features (Radchenko and Elmes 2010). However, unfortunately the diversity from Indian Himalaya is very poorly represented in this census. There are reasons galore for this, but the most significant being lack of material collected from this region. The diversification of the major ant lineages, as elucidated by Moreau et al. (2006), occurred from the beginning of the early Paleocene to the late Cretaceous, 60 to 100 million years ago in the age of angiosperms. Interestingly, the same time span coincides with the geological history of Himalaya; the initial mountain building processes were underway about 70 million years ago when the North moving Indo-Australian plate collided with the Eurasian plate, followed by a second phase of mountain development about 65 million years ago. Upliftment of Himalaya as an isolation barrier has led to lot of endemism, thus many species groups which remain concentrated here, diversified in this region only (Bharti 2008a, b, 2011; Bharti and Sharma 2011; Radchenko and Elmes 2001, 2010). In last decade or so, the author and his team have started exploring Himalayan fauna and as per expectations the region has quite a number of undescribed/unnoticed species (reasonably unique too) which can contribute a lot to understanding the *Myrmica* of old world and would provide an insight into many unresolved questions.

Although the species groups in *Myrmica* as created by Radchenko and Elmes (Radchenko and Elmes 2001, 2010) are arbitrary divisions they seem to be correct as verified by molecular studies (Jansen et al. 2009, 2010) and appear to be monophyletic. *Myrmica elmesi* sp. n. which is described here, belongs to the *pachei* group, which is characterized by an alitrunk dorsum atleast partly with transverse rugosity; scape gradually though distinctly curved at base, not angled, with no trace of lobe or carina; anterior clypeal margin rounded or slightly prominent with no medial notch; petiole with a relatively short peduncle (Radchenko and Elmes 2010). Earlier this group was considered rare and unusual however Radchenko and Elmes (2008, 2009) while examining *Myrmica* fauna of China have found that it is more diverse than previously expected. Now it is represented by 14 species which are found in Himalaya, south-western and southern China. It is supposed that like the *rugosa* group, the *pachei* group is derived from a *ritae* like ancestor that adapted to somewhat cooler temperate, open conditions that are found at slightly lower altitudes on more northern mountains in the Oriental region, where local isolation has led to a variety of local forms (Radchenko and Elmes 2010). *Myrmica elmesi* sp. n. is quite different from previously described species of *pachei* group; it was collected from the transitional zone of Himalaya and is described with notes on its ecology. This gains significance in the sense that ecology of most of the old world *Myrmica* is either unknown or poorly known and furthermore this is the fourth species of the diverse *pachei* group found in Himalaya.

## Materials and methods

The specimens were collected through winkler’s extractor and were preserved in 70% alcohol. Then the mounted material was analyzed on Nikon SMZ-1500 stereo zoom microscope. For digital images, MP evolution digital camera was used on same microscope with Auto-Montage (Syncroscopy, Division of Synoptics, Ltd) software. Later, images were cleaned as per requirement with Adobe Photoshop CS.

We used measurements and indices proposed by (Radchenko and Elmes (1998, 2010):

HL length of head in dorsal view, measured in a straight line from the anterior point of median clypeal margin to mid-point of the occipital margin.

HW maximum width of the head in dorsal view behind the eyes.

FW minimum width of the frons between the frontal carinae.

FLW maximum width between the external/outer borders of the frontal lobes.

SL maximum straight-line length of antennal scape in profile.

AL diagonal length of the alitrunk seen in profile, from the neck shield to the posterior margin of propodeal lobes (workers) and from the anterio-dorsal point of alitrunk to posterior margin of propodeal lobes (queens).

AH Height of alitrunk, measured from upper level of mesonotum perpendiculary to the level of lower margin of mesopleuron (queens and males).

PL maximum length of petiole from above, in dorsal view, measured from the posterolateral margin of petiole to the articulation with propodeum, the petiole should be positioned so that measured points lay on the same plane.

PPL maximum length of post-petiole in dorsal view between its visible anterior and posterior margins.

PW maximum width of petiole in dorsal view.

PPW maximum width of postpetiole from above/in its dorsal view.

PH maximum height of petiole in profile, measured from the uppermost point of petiolar node perpendicularly to the imaginary line between anteroventral(just behind the subpetiolar process) and posteroventral points of petiole.

PPH maximum height of postpetiole in profile from the uppermost to the lowermost point, measured perpendicularly to the tergo-sternal suture.

ESL maximum length of propodeal spine in profile, measured along the spine from its tip to the deepest point of the propodeal constriction at the base of the spine.

ESD distance between tips of spines from above/in dorsal view.

PNW maximum width of pronotum from above/in dorsal view.

**Indices used**

Cephalic CI = HL/HW

Frontal FI = FW/HW

Frontal lobe FLI = FLW/FW

Scape (1) SI1 = SL/HL

Scape (2) SI2 = SL/HW

Petiole (1) PI 1 = PL/PH

Petiole (2) PI2 = PL/HW

Petiole (3) PI3 = PW/HW

Post-petiole (1) PPI1 = PPL/PPH

Post-petiole (2) PPI2 = PPH/PPW

Post-petiole (3) PPI3 = PPW/PW

Post-petiole (4) PPI4 = PPW/HW

Spine length ESLI = ESL/HW

Spine width ESDI = ESD/ESL

## Results

### Myrmica elmesi sp. n.

urn:lsid:zoobank.org:act:F5D15679-304A-475B-A2F8-5F60FF0B4022

http://species-id.net/wiki/Myrmica_elmesi

Figs 1–3 Table 1

#### Type locality.

India, Jammu and Kashmir, Machedi, 32.72364°N, 75.669464°E, 2000 meters above mean sea level. Collected 3rd August, 2008 by Yash Paul Sharma.

#### Holotype.

Worker from type locality, triangle mounted. Deposited in Department of Zoology (Dr Himender Bharti’s Collections- DST-YEG-562), Punjabi University Patiala, India.

#### Paratypes.

 1 worker (with same data as of holotype) and 10 workers from India, Jammu and Kashmir, Sarthal, 32.812947°N, 75.762503°E, 2200 metres above mean sea level, all paratype triangle mounted. Collected 15th June, 2009 by Yash Paul Sharma. Deposited in Department of Zoology (Dr. Himender Bharti’s Collections-DST-YEG-292, 293, 294, 296, 297, 298, 299, 300, 344, 345, 561) Punjabi University Patiala, India. One of the paratypes will be deposited at Natural History Museum, London.

#### Description

 (worker).Worker measurements: FLW 0.40, FW 0.39, HL 1.10, HW 0.88, SL 0.94, PL 0.51, PPL 0.41, PW 0.24, PPW 0.34, PH 0.30, PPH 0.34, AL 1.57, TL 5.26.

Head distinctly longer than broad, with parallel sides and straight occipital margin. Mandibles with 9 teeth (apical and preapical ones are the largest), masticatory margin black, whole mandible finely longitudinally costulate, rugulose and punctated. Clypeus convex, longitudinally rugulose, anterior clypeal margin prominent and rounded medially, spaces between rugae minutely punctated but appear shiny. Frontal triangle somewhat deep, smooth and shiny. Frontal carinae short, partially cover the condylar bulb, almost straight, curving outwards to merge with rugae that surround antennal sockets. Antennae 12 segmented, funicular segments densely punctated, but two basal segments finely punctated; scape slender, narrow, weakly curved at base, without any trace of angle or carina, running towards apex just extending beyond upper margin of head; antennae with oblique short semi-erect hairs, with pubescence developed only on 3 apical segments, antennal club 3 segmented. Eyes large, situated slightly below the midlength of head. Head dorsum longitudinally rugose, 11 rugae between frontal carinae at the level of eyes, occiput with reticulate sculpture, opaque; anterior clypeal margin fringed with setae; head, clypeus and mandibles with long hairs.

Promesonotal dorsum feebly convex, forming regular arch, not saddle shaped, promesonotal suture indistinct. Promesonotum transversally sinuously rugose in dorsal view, pronotum transversally striate laterally; metanotal groove broad, deep and longitudinally striate. Propodeal dorsum longitudinally striated, declivity smooth and highly polished, mesonotum and propodeum longitudinally striate laterally, propodeal lobes rounded apically; propodeal spines long, sharp, projected upward and divergent. Tibiae of hind and middle legs with well-developed pectinate spur.Petiole with short anterior peduncle, petiolar dorsum rounded; whole petiole punctated and finely reticulated, appears dull. Postpetiole slightly longer than broad, finely punctated and longitudinally striated. In profile, petiole high and narrow with a short tooth like subpetiolar process. Alitrunk, petiole and postpetiole with long hairs, except for propodeum.

Gaster smooth, highly polished and shiny, with long, erect hairs.

**Figures 1–3.**
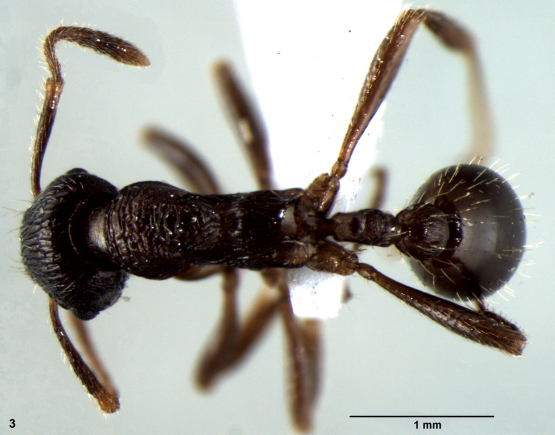
**1** Head of *Myrmica elmesi* sp. n., dorsal view **2** Body of *Myrmica elmesi* sp. n., lateral view **3** Body of *Myrmica elmesi* sp. n., dorsal view.

**Table 1.** The mean, standard deviation, minimum and maximum values (in mm) of the measurements and indices made on samples of species (workers). The measurement codes are as indicated in the text and the numbers of individuals measured are given in parenthesis.<br/>

*Measurements*		*Holotype*	*Workers (12)*								
			*Mean±SD*	*Min*	*Max*	*Indices*		*Holotype*	*Mean±SD*	*Min*	*Max*
	HL	1.10	1.09±0.07	0.89	1.17		CI	1.25	1.24±0.08	1.07	1.30
	HW	0.88	0.87±0.04	0.81	0.97		FI	0.44	0.44±0.01	0.41	0.45
	SL	0.94	0.91±0.04	0.85	1.00		FLI	1.03	1.05±0.01	1.03	1.08
	PL	0.51	0.49±0.03	0.42	0.52		PI1	1.70	1.64±0.08	1.50	1.78
	PH	0.30	0.30±0.01	0.28	0.32		PI2	0.58	0.56±0.03	0.52	0.60
	PW	0.24	0.24±0.01	0.23	0.27		PI3	0.27	0.28±0.01	0.26	0.30
	PPL	0.41	0.40±0.02	0.36	0.43		PPI1	1.21	1.14±0.05	1.06	1.21
	PPH	0.34	0.35±0.02	0.33	0.39		PPI2	0.97	0.96±0.02	0.92	1.00
	PPW	0.35	0.37±0.02	0.35	0.40		PPI3	1.46	1.53±0.07	1.44	1.63
	FLW	0.40	0.40±0.02	0.36	0.43		PPI4	0.40	0.42±0.01	0.40	0.44
	FW	0.39	0.38±0.02	0.35	0.40		SI1	0.85	0.84±0.04	0.80	0.96
	ESL	0.24	0.22±0.02	0.19	0.26		SI2	1.07	1.03±0.04	0.93	1.09
	AL	1.57	1.51±0.07	1.33	1.57		ESLI	0.27	0.26±0.03	0.20	0.31
	ESD	0.40	0.37±0.03	0.33	0.41		ESDI	1.67	1.67±0.20	1.27	1.95
	PNW	0.65	0.64±0.02	0.60	0.67						

#### Etymology.

The species is dedicated to Dr Graham Elmes for his contribution to the investigation of genus *Myrmica*.

#### Distribution and habitat.

North-west Himalaya (India, Jammu and Kashmir).

#### Ecology.

 Species has been collected from leaf litter in both the habitats. The collection site at Machedi has a patchy *Cedrus* forest along with agricultural land surrounding the site; moreover the area has lot of anthropogenic activities with a dry type of environment (mean temperature during collection period 32°C, relative humidity 36.62%, annual rain fall 970mm and thickness of leaf litter 2.1cm). The collection site at Sarthal has dense *Cedrus* forest with abundant leaf litter, no agricultural land, it remains snow clad from November to beginning of March and has very limited anthropogenic activities with only nomads visiting the area (mean temperature during collection period 22°C, relative humidity 66.38%, annual rain fall 1476mm and thickness of leaf litter 3.9cm) with comparatively wet environment.

This zone where the species is distributed is a transitional zone between sub temperate and temperate Himalaya and geographically it penetrates in to the Palearctic zone (whose boundary in Southern Asia is largely altitudinal, where an altitude of 2000–2500 meters above mean sea level forms the boundary between Palearctic and Indo-Malayan ecozones). Besides, Himalayan ecology is temperature-dependent. The snow line occurs at an average of 6000 meters above mean sea level and the timber line at an average of 3000 meters (the highest altitude at which the forest ends). With this sort of environment, the micro-climate plays an important role for ants like *Myrmica* which prefer to live under stones or in rare cases in leaf litter, as the soil temperature is comparatively higher to ambient temperature in these habitats (Bharti 2008b).

#### Conservation status.

Yet to be ascertained, but population level could be low as collected from leaf litter only.

#### Discussion.

*Myrmica elmesi* sp. n. (Figs 1–3) which belongs to *pachei* group, and is significantly different from all other described species of this group due to its transversally striated promesonotum (seen in dorsal view), longitudinally striated propodeal dorsum and PI1 1.64. In all the known species of *pache*i group hitherto, either the pronotal and propodeal dorsum are transversally striated, or the whole alitrunk dorsum has transverse rugae, or the mesonotal and propodeal dorsum has coarse/fine transverse rugosity. A PI1> 1.5 has been recorded in only one other species of the *pachei* group (i.e. PI1=1.68 for *Myrmica weii* Radchenko & Zhou, 2008 in Radchenko et al. 2008). *Myrmica elmesi* can be placed in to the recent key provided by Radchenko and Elmes (2010, page 736) in the following way 1-2-3 and at couplet 3;

**Table:** 

3	Promesonotal dorsum with transverse rugae, propodeal dorsum with fine longitudinal striations	*Myrmica elmesi* sp. n.
–	Either whole alitrunk dorsum with transverse rugae or pronotal and propodeal dorsum with transverse rugae	4
4	Pronotal and propodeal dorsum with transverse rugae, other part of alitrunk dorsum with longitudinal rugosity and reticulation	*Myrmica varisculpta* Radchenko & Rigato, 2009 in Radchenko and Elmes 2009
–	Whole alitrunk dorsum with transverse rugae	5

(Then couplet 5 onwards *Myrmica pachei* Forel, 1906, *Myrmica inezae* Forel, 1902 and *Myrmica villosa* Radchenko & Elmes, 1999 can be keyed out as given in the above mentioned key of Radchenko and Elmes 2010).

## Supplementary Material

XML Treatment for Myrmica elmesi
